# *Rhodococcus aromaticivorans* sp. nov., an *o*-xylene degrading bacterium, and evidence supporting reclassification of *Rhodococcus jostii* RHA1

**DOI:** 10.1371/journal.pone.0337194

**Published:** 2025-12-02

**Authors:** Neak Muhammad, Jehyun Jeon, Eungbin Kim, Dockyu Kim, Yung Mi Lee

**Affiliations:** 1 Division of Life Sciences, Korea Polar Research Institute, Incheon, Republic of Korea; 2 Department of Systems Biology, Yonsei University, Seoul, Republic of Korea; Irkutsk State University: Irkutskij gosudarstvennyj universitet, RUSSIAN FEDERATION

## Abstract

A Gram-positive, aerobic, non-motile bacterium with a rod-coccus shape, designated DK17^T^, was isolated from a crude oil-contaminated soil and identified as a member of the genus *Rhodococcus* based on 16S rRNA gene analysis, showing highest similarity (99.93%) to *Rhodococcus jostii* DSM 44719^T^. However, average nucleotide identity (ANI) and digital DNA-DNA hybridization (dDDH) values between strain DK17^T^ and type strains within the genus *Rhodococcus* were below the species delineation thresholds of 95% and 70%, respectively. In contrast, DK17ᵀ exhibited ANI and dDDH values over 99% and 92%, respectively, with *R. jostii* RHA1. Comparative genomic analysis revealed that DK17^T^ and RHA1 shared 93.5% of genes, while RHA1 and *R*. *jostii* NBRC 16295^T^ shared only 78.6%, indicating a closer relationship between DK17^T^ and RHA1. Both strains possess large genomes (~9.5–9.7 Mb) comprising a linear chromosome and multiple plasmids, and encode multiple dioxygenases and secondary metabolite biosynthetic gene clusters. *In vitro* assays confirmed *o*-xylene degradation by both DK17^T^ and *R. jostii* RHA1, consistent with the presence of the *akb* gene cluster. Both strains shared C_16:0_ as a major fatty acid and menaquinone-8 (H2) as the dominant quinone. Based on genomic, phenotypic, and chemotaxonomic data, DK17^T^ (=KCCM 90599^T^ = InaCC B1662^T^) is proposed as a novel species, *Rhodococcus aromaticivorans* sp. nov., and *R. jostii* RHA1 is reclassified as a member of the same species.

## 1 Introduction

The genus *Rhodococcus*, belonging to the family *Nocardiaceae*, order *Mycobacteriales*, and phylum *Actinomycetota*, was first proposed in 1891 with *Rhodococcus rhodochrous* designated as the type species [1,2]. According to LPSN, the genus *Rhodococcus* currently includes 59 validly published species (https://lpsn.dsmz.de/genus/rhodococcus). However, recent phylogenomic analyses suggest that some of these species may belong to different genera, with several proposed to be reclassified into *Rhodococcoides* and *Prescottella* [3,4], indicating that the taxonomy of *Rhodococcus* is still under revision.

Members of the genus *Rhodococcus* are Gram-positive and non-motile *Actinomycetota* that display notable morphological variability depending on growth conditions and are characterized by menaquinone (MK)-8(H2) as their primary isoprenoid quinone [4]. *Rhodococcus* strains have been isolated from a wide array of environments, including soil, rocks, groundwater, marine sediments, animals, and plants, and are recognized for their extensive physiological versatility [5–11]. This ecological versatility is likely linked to the genomic diversity among *Rhodococcus* species, with genome sizes ranging from 3.71 to 10.91 Mb (https://www.ncbi.nlm.nih.gov/datasets/genome/?taxon=1827). Such differences in genome size are presumed to contribute to the broad metabolic capabilities of *Rhodococcus* species, facilitating their adaptation to diverse ecological niches [11].

The metabolic versatility of *Rhodococcus* species enables them to degrade a wide range of natural and synthetic compounds, including both aliphatic and aromatic hydrocarbons and various environmental pollutants [12]. For example, *Rhodococcus* sp. RHA1 can metabolize a variety of aromatic compounds, notably polychlorinated biphenyls (PCBs) [13]. *R. oxybenzonivorans,* isolated from a stream flowing through an industrial complex, can degrade aliphatic and aromatic hydrocarbons, including benzophenone-3, naphthalene, phenanthrene, and hexadecane [14]. Similarly, *R. chlorophenolicus* thrives in PCB-contaminated sediments, utilizing biphenyl as a carbon source, while *R. phenolicus* degrades compounds such as phenol, chlorobenzene, and dichlorobenzene [15,16]. The presence of mycolic acids in their cell walls enhances their ability to degrade hydrophobic pollutants, further supporting their role in the breakdown of persistent organic contaminants [12,17]. In particular, *R. opacus* R7 is well-known for its ability to degrade both naphthalene and *o*-xylene [18–20]. Recent studies have further demonstrated that *Rhodococcus* strains are capable of degrading polycyclic aromatic hydrocarbons (PAHs) and dibenzofuran, with biosurfactant production and high cell-surface hydrophobicity enhancing pollutant uptake and bioavailability [21,22]. Collections of alkanotrophic *Rhodococcus* strains have also revealed broad substrate spectra, including aromatic, N-heterocyclic, and substituted aromatic compounds, highlighting their high efficiency in hydrocarbon-contaminated soil remediation [21,23]. The substantial catabolic diversity observed in several *Rhodococcus* strains underscores their significant potential for environmental applications, particularly in bioremediation [14,16,24,25].

The large genomes and megaplasmids of *Rhodococcus* strains confer substantial metabolic versatility, supporting pollutant degradation and the production of secondary metabolites such as antimicrobial agents, bioflocculants, and biosurfactants. The first sequenced *Rhodococcus* genome, from *R. jostii* RHA1, spans 9.7 Mbp, including a 7.8 Mbp chromosome and three plasmids, encoding genes for the degradation of diverse compounds [13]. This strain shows extraordinary catabolic diversity including an ability to metabolize halogenated hydrocarbons, ketoaromatic compounds and steroids [13]. *R. erythropolis* PR4, capable of degrading long-chain alkanes, possesses multiple monooxygenase genes [24,26], while *R*. *opacus* R7 is equipped with genes that target a broad range of aliphatic and aromatic hydrocarbons [18,20,27]. The genomic analysis of *R. oxybenzonivorans* identified 89 monooxygenase and 62 dioxygenase genes, suggesting that this strain has a versatile ability to biodegrade various organic pollutants because the aerobic biodegradation of organic pollutants is generally initiated by oxygenases such as monooxygenase or dioxygenase [14]. These examples highlight the remarkable metabolic potential of *Rhodococcus* species, underscoring the significance of continued efforts to isolate and investigate novel strains from diverse ecological niches.

*Rhodococcus* strain DK17^T^, isolated from crude oil-contaminated soil in Korea, utilizes *o*-xylene as its sole carbon and energy source, exhibiting a doubling time of 2.4 hours. Although it cannot grow on *m*- or *p*-xylenes, DK17^T^ is capable of degrading a variety of alkylbenzenes (e.g., toluene, ethylbenzene, isopropylbenzene), benzene derivatives (e.g., *n*-propyl- to *n*-hexylbenzenes, *p*-toluate, and *m*- and *p*-hydroxybenzoates) and phthalate [28–30]. Genome analysis of DK17^T^ revealed the presence of three linear megaplasmids (~399 kb pDK1, ~ 318 kb pDK2, and ~779 kb pDK3), and the *akb* operon, known to catalyze *o*-xylene degradation [28], is located on pDK2 [31]. This operon encodes enzymes involved in alkylbenzene catabolism, including two sets of large and small subunits of *o*-xylene dioxygenase (*akbA1a*-*akbA2a* and *akbA1b*-*akbA2b*), a ferredoxin (*akbA3*) and ferredoxin reductase (*akbA4*), a dehydrogenase (*akbB*) and a series of *meta*-cleavage pathway enzymes including *meta*-cleavage 2,3-dioxygenase (*akbC*), hydrolase (*akbD*), hydratase (*akbE*), and aldolase (*akbF*) [32]. Functional characterization through recombinant expression in *E. coli* and gene expression profiling confirmed the conversion of *o*-xylene to *cis*-3,4-dihydrodiol by AkbA1A2A3A4, subsequent transformation to 3,4-dimethylcatechol by AkbB, and ring-cleavage by AkbC [33,34], thereby establishing the proposed degradative pathway toward tricarboxylic acid (TCA) cycle intermediates.

Despite the advancements in genome-based taxonomy, the precise classification of industrially significant *Rhodococcus* strains, including DK17^T^, remains unresolved. Recent studies suggest that certain strains, such as DK17^T^ and RHA1, may belong to a distinct group within the genus, referred to as Cluster C or subgenus *Anisorhodococcus*, indicating ongoing challenges in defining species boundaries [3,35,36]. To address this, we conducted a comparative analysis of physiological and genomic characteristics of DK17^T^ strain in relation to closely related *Rhodococcus* species, with the aim of clarifying its taxonomic position and evaluating its biotechnological potential in environmental applications and secondary metabolite production.

## 2 Materials and methods

### 2.1 Isolation and culturing conditions

Soil samples were collected in 1999 from an open access area within the Yeocheon Industrial Complex (Yeosu, South Korea). At the time of the sampling, the site was publicly accessible and no specific permits were required. Strain DK17 was isolated from *o*-xylene-degrading enrichment cultures using minimal salts basal (MSB) medium [37], with *o*-xylene supplied in the vapor phase and incubation at 30°C with shaking [28]. A 5 mL aliquot of the enriched culture, free of soil particles, was transferred to fresh MSB supplemented with *o*-xylene, followed by streaking onto MSB with 20 mM glucose (MSB + Glu) or Reasoner’s 2A (R2A) agar. The strain that grew well on both media was designated DK17^T^. Pure colonies were preserved in 20% glycerol at −80°C. Strain DK17^T^ was deposited to the Korea Culture Center of Microorganisms (KCCM) and the Indonesian Culture Collection (InaCC).

### 2.2 Sequencing of 16S rRNA gene and phylogenetic analysis

Genomic DNA from strain DK17^T^, grown on MSB + Glu, was extracted by using the genomic DNA preparation reagent (PrepMan Ultra Sample Preparation Reagent, Thermo Fisher Scientific). The 16S rRNA gene was amplified using the universal primers, 27F and 1492R [38]. PCR products were purified using a PCR purification kit and sequenced using primers, 785F and 907R [38]. The 16S rRNA gene sequences were assembled using Vector NTI software and searched in the EzBioCloud database for matching sequences [39]. The sequences downloaded from EzBioCloud were aligned and trimmed using BioEdit software. Three types of phylogenetic trees were constructed utilizing the molecular evolutionary genetics analysis (MEGA X) software [40]: neighbor-joining (NJ) [41], maximum likelihood algorithms (ML) [42] and the maximum parsimony (MP) method [43]. All the phylogenetic trees were evaluated using 1,000 bootstrap iterations, with *Corynebacterium diphtheriae* NCTC 11397^T^ (X84248) as the outgroup.

### 2.3 Genome sequencing, assembly, and annotation

The complete genome sequence of strain DK17^T^ was sequenced using a combination of Oxford Nanopore Technology (ONT) and Illumina NovaSeq, as previously described by Jeon et al. (2025) [31]. Raw reads were quality-filtered using NanoFilt (v2.6.0) with parameters of -q 10 and -l 5000 for Nanopore and Trimmomatic (v0.21.0) for Illumina [44,45]. Hybrid assembly was performed using Unicycler (v0.4.8-beta) [46], and genome completeness and contamination were assessed using CheckM (v1.2.2) [47]. Annotation was conducted with the NCBI Prokaryotic Genome Annotation Pipeline [48]. Functional analyses including detection of biosynthetic gene clusters (BGCs), were performed using RAST [49] and antiSMASH (v7.0) [50] with default parameters.

### 2.4 Genome-based relatedness and functional features comparison

For phylogenomic analysis, multiple sequence alignments of the concatenated 120 ubiquitous single-copy proteins were performed using GTDB-Tk [51]. Two datasets were analyzed: one comprising thirty-two available type strain genomes within the genus *Rhodococcus* along with DK17^T^, *R. jostii* RHA1 and *R. opacus* R7, and the other including six additional genomes designated as *R. jostii* (*R. jostii* NPDC059932, *R. jostii* NPDC059950, *R. jostii* NPDC127600, *R. jostii* IEGM 60, *R. jostii* DSM 44719^T,^ and *R. jostii* CCM 4760). Phylogenomic trees were reconstructed based on 1,000 sets of sequence replications using the maximum likelihood algorithm by MEGA X [40,42,52]. To assess genome-based similarity among 35 available *Rhodococcus* genomes, pairwise genome-based relatedness was estimated by calculating average nucleotide identity (ANI) using EzBioCloud (www.ezbiocloud.net/tools/ani) [39]. Digital DNA–DNA hybridization (dDDH) among genomes that showed > 94% ANI values with DK17^T^, including *R*. *jositi* RHA1, *R*. *opacus* ATCC 51881^T^, *R*. *opacus* R7, and *R*. *wratislaviensis* NBRC 100605^T^, as well as the type strain R. *jostii* NBRC 16295^T^ (genome-sequenced strain, equivalent to the type strain DSM 44719^T^) was calculated using the genome-to-genome distance calculator (GGDC) (version 3.0, DSMZ; https://ggdc.dsmz.de/ggdc.php#) [39,53]. Core gene sharing analysis and comparative assessment of the genomic basis for *o*-xylene degradation and synthesis of secondary metabolites were performed using genomes that showed >94% ANI values with DK17^T^, including *R. jostii* RHA1, *R*. *opacus* ATCC 51881^T^, *R*. *opacus* R7, and *R*. *wratislaviensis* NBRC 100605^T^, as well as the type strain *R*. *jostii* NBRC 16295^T^. Comparative genome analysis was performed by sequence clustering using CD-HIT (v4.8.1) [54] with a 70% sequence identity threshold to investigate core genes between genomes. UpSet plot was visualized using UpSetR package (v1.4.0) in R [55].

### 2.5 Physiological and biochemical characterization

To determine physiological and biochemical characteristics, strains DK17^T^, *R. jostii* RHA1, and *R. opacus* KCTC 9811^T^ (=ATCC 51881^T^) were cultivated on R2A agar. Gram reaction was performed using Gram-stain kit (Sigma). The morphology of the cells was examined using transmission electron microscopy (TEM; CM200, Philips). For TEM analysis, the cells of strains DK17^T^ and *R. jostii* RHA1 were negatively stained with 2.0% uranyl acetate on a carbon-coated copper grid [56]. Motility of strains DK17^T^, *R. jostii* RHA1, and *R. opacus* KCTC 9811^T^ was determined by the observation of growth after inoculation in the R2A liquid medium with 0.4% agar. The optimal temperature for growth of strains DK17^T^, *R. jostii* RHA1, and *R. opacus* KCTC 9811^T^ was determined by culturing strains on R2A at different temperatures (0, 4, 10, 15, 20, 25, 30, and 37°C). NaCl tolerance tests for all three strains were conducted on R2A agar supplemented with varying concentration of NaCl (0, 0.5, 1, 2, 3, 4, 5, 6, 7, 8, and 9% w/v) [57]. Growth on agar plate was evaluated based on visible colony formation after incubation.

The optimal pH for growth for all three strains was determined in R2A liquid medium. The pH was adjusted using the following buffering systems; Na_2_HPO_4_-buffered citric acid, pH 4.0–5.0; MES, pH 5.5–6.0; MOPS, pH 6.5–7.0; AMPD, pH 8.0–9.5; CAPS, pH 10.0. The growth of each culture was assessed by measuring optical density at 600 nm (EnVision plate reader, PerkinElmer) [56]. The growth under anaerobic culture condition was determined using a jar containing an AnaeroPak (Mitsubishi Gas Chemical). Biochemical characterization for strains DK17^T^, *R. jostii* RHA1, and *R. opacus* KCTC 9811^T^ were determined. Catalase activity was tested using 3% (v/v) hydrogen peroxide and oxidase activity was assessed with 1% (w/v) tetramethyl-*p*-phenylenediamine dihydrochloride [58,59]. The ability to hydrolyze casein, chitin, hypoxanthine, starch, xanthine, and Tweens 20, 40, 60, and 80 was evaluated on R2A agar [60]. Biochemical characteristics and enzymatic activities were determined using API ZYM and API 20NE following the manufacturer’s instructions (bioMérieux).

### 2.6 Chemotaxonomic characterization

To determine the cellular fatty acid profile, cells from strains DK17^T^, *R. jostii* RHA1, *R. opacus* KCTC 9811^T^ were collected from R2A agar after incubation at 30°C for two days. The fatty acids were extracted following the MIDI protocol (Sherlock Microbial Identification System version 6.0) and analyzed using a gas chromatography (GC) system at KCCM (Seoul, Korea) [61]. Quinones were extracted as described by Collins and Jones and analyzed by high-performance liquid chromatography (HPLC) at KCCM [62].

### 2.7 *In vitro* assay for *o*-xylene degradation

For the degradation of *o*-xylene, freshly prepared cultures of DK17^T^, *R. jostii* RHA1, and *R. opacus* KCTC 9811^T^, were transferred to MSB agar plates, with *o*-xylene supplied as the sole carbon and energy source. *o*-Xylene in liquid form was added to a glass bulb with a cotton stopper and directly supplied to the strains as a vaporized gas in an airtight container [28]. As a control, the strains were also transferred to MSB agar without *o*-xylene. The plates were incubated at 30°C for three days, and after incubation, growth on the MSB media was observed and compared between the presence and absence of *o*-xylene.

## 3 Results and discussion

### 3.1 16S rRNA gene-based phylogeny

The phylogenetic analysis using 16S rRNA gene sequence confirmed that strain DK17^T^ is affiliated to the genus *Rhodococcus,* showing the highest sequence similarities with *R. jostii* DSM 44719^T^ (99.93%), *R. koreensis* DSM 44489^T^ (99.36%), *R. oxybenzonivorans* S2-17^T^ (99.21%), and *R. percolatus* MBS1^T^ (99.06%). The 16S rRNA gene similarity values between strain DK17^T^ and closely related strains exceeded the cutoff values of species discrimination (98.7%) [63]. Phylogenetic trees based on 16S rRNA gene sequences showed a monophyletic clustering of the strain DK17^T^ with reference strains *R. jostii* DSM 44719^T^, *R. jostii* RHA1, and *R. koreensis* DSM 44498^T^ (Figure S1 in S1 File).

### 3.2 Genome-based relationship and comparison of general genomic features

**Fig 1:**
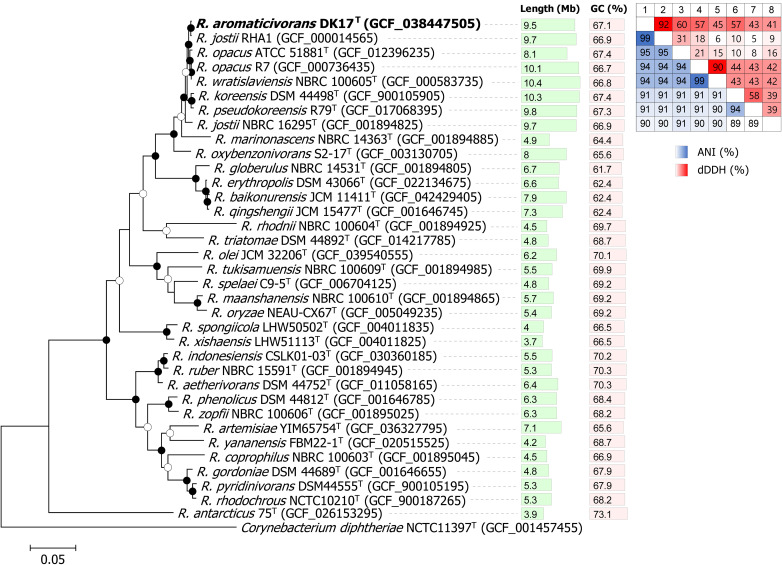
Maximum likelihood phylogenomic tree inferred from concatenated alignments of 120 single-copy amino acid sequences in Genome Taxonomy Database (GTDB). Genomic sequences were obtained from the NCBI RefSeq database under the corresponding assembly accession number. Bootstrap values are represented by the nodes as filled circle (>90%) and empty circle (>50%). *Corynebacterium diphtheria* NCTC11397^T^ (GCF_001457455) was used as an outgroup. Scale bar, 0.05 substitutions per amino acid position. Average nucleotide identity (ANI) and digital DNA-DNA hybridization (dDDH) were calculated for the following genomes: 1. DK17^T^, 2. *R. jostii* RHA1, 3. *R. opacus* ATCC 51881^T^, 4. *R. opacus* R7, 5. *R. wratislaviensis* NBRC100605^T^, 6. *R. koreensis* DSM 44498^T^, 7. *R. pseudokoreensis* R79^T^, 8. *R. jostii* NBRC 16295^T^.

The CheckM analysis indicated a value of 99.59% completeness and 2.01% contamination, demonstrating the high quality of DK17^T^ genome (Table S1 in S2 File). The 16S rRNA gene sequences of strain DK17^T^ (1,430 bp), determined by direct sequencing, were identical to those retrieved from its genome sequences. A genome-based phylogenetic tree demonstrated a robust clustering of strain DK17^T^ and *R. jostii* RHA1 within the genus *Rhodococcus*, supporting their close evolutionary relationship while *R. jostii* NBRC 16295^T^ was more distantly related to RHA1 (Fig 1). In addition, four genomes deposited under the name *R. jostii* (*R. jostii* NPDC059932, *R. jostii* NPDC059950, *R. jostii* NPDC127600, and *R. jostii* IEGM 60) also clustered with DK17^T^ and *R. jostii* RHA1 (Figure S2 in S1 File), suggesting potential misidentification of these strains. To further confirm the taxonomic position and novelty of strain DK17^T^, ANI and dDDH values were calculated against the closely related strains forming a monophyletic clade in the phylogenomic tree. The ANI and dDDH values between strain DK17^T^ and closely related strains in the phylogenomic tree, except for *R. jostii* RHA1, were below the standard cut-off values for speciation, with ANI values under 95–96% and dDDH values below 70% (Fig 1, Table S2 in S2 Table) [53,64]. This suggests that DK17^T^ can be considered a novel species. In contrast, the ANI and dDDH values between strain DK17^T^ and *R. jostii* RHA1 were 99% and 92%, respectively, supporting their classification as the same species. In addition, four genomes deposited under the name *R. jostii* (*R. jostii* NPDC059932, NPDC059950, NPDC127600, and IEGM 60) showed >95% ANI values with DK17^T^, whereas their ANI values with the *R. jostii* type strain genomes were approximately 90% (Figure S2 in S1 File). Previously, *Rhodococcus* strain RHA1 was classified as a bona fide member of the species *R. jostii* based on a combination of phenotypic characteristics and DNA-DNA relatedness, which was manually determined to exceed 87%, well above the 70% threshold for species delineation [65]. However, this study re-evaluates the classification of *Rhodococcus* strain RHA1 using a comprehensive genome-based approach, providing a more precise and robust basis that refines prior classifications. Our analysis showed that the ANI values between *R. jostii* RHA1 and the *R. jostii* type strains, *R. jostii* DSM 44719^T^ and *R. jostii* NBRC 16295^T^ were 90% indicating that *R. jostii* RHA1 is distinct from the established *R. jostii* type strains. Consistent with recent analyses of intrageneric structure within *Rhodococcus*, DK17^T^ and RHA1 clearly fall within Cluster C, which corresponds to the proposed subgenus *Anisorhodococcus* [35,36]. This cluster is phylogenetically distinct from the one containing the authentic *R. jostii* type strain, further supporting that RHA1 and DK17^T^ should not be classified as *R. jostii*. Furthermore, our analysis showed that *R. opacus* R7 and *R. wratislaviensis* NBRC 100605^T^ belong to the same species, with a pairwise ANI of 98.7% and a dDDH value of 90% (Fig 1, Table S2 in S2 Table).

The total genome size of strain DK17^T^ is 9.5 Mb, with a GC content of 67.1% (Fig 1, Table S1 in S2 File). The genome sizes of *Rhodococcus* strains that formed a monophyletic clade with DK17^T^ in the phylogenomic tree ranged from 8.1 to 10.4 Mb, which is larger than those in other clades (3.7–7.9 Mb) (Fig 1). Notably, our phylogenomic analysis shows that RHA1 is well separated from the *R. jostii* type strain, consistent with the observations reported by Sangal et al. (2019) [35]. The GC contents of strains forming a monophyletic clade with DK17^T^ ranged from 66.7 to 67.4%, which is consistent within genus *Rhodococcus* (61.7–73.1%) (Fig 1). The genome of strain DK17^T^ comprises one linear chromosome, three linear mega-plasmids, and two circular plasmids (Table S1 in S2 File). *Rhodococcus jostii* RHA1 and *R*. *opacus* R7 possess three and five linear plasmids, respectively (Table S1 in S2 File). In contrast, plasmids in *R*. *opacus* ATCC 51881^T^, *R*. *wratislaviensis* NBRC 100605^T^, and *R*. *jostii* NBRC 16295^T^ were not fully determined, partially due to the low sequencing depths.

**Fig 2:**
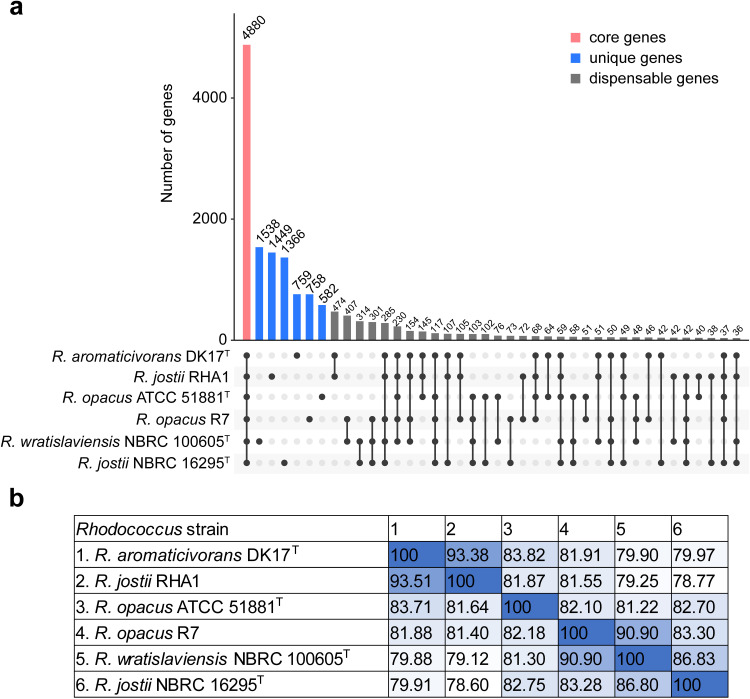
Comparative genome analysis of DK17^T^ with closely related *Rhodococcus* strains. **(a)** UpSet plot represents the core, unique, and accessory genes between *Rhodococcus* genomes. **(b)** The proportion of shared genes.

Identification of core and unique genes among strain DK17^T^ and five *Rhodococcus* strains, *R. jostii* RHA1, *R. opacus* R7, *R. opacus* ATCC 51881^T^, *R. wratislaviensis* NBRC 100605^T^, and *R. jostii* NBRC 16295^T^, revealed that all strains share 4,880 core genes, with 759 unique genes identified in strain DK17^T^ (Fig 2). Notably, DK17^T^ shared 93.4–93.5% of its genes with *R. jostii* RHA1 while only 79.9–80.0% with *R. jostii* NBRC 16295^T^. In addition, the proportion of shared genes between *R. jostii* RHA1 and *R. jostii* NBRC 16295^T^ is 78.6–78.8%. This suggests that *R. jostii* RHA1 and *R. jostii* NBRC 16295^T^ are distinct taxa, while DK17^T^ and RHA1 are more closely related.

### 3.3 Physiological and biochemical characterization

Strain DK17^T^ was Gram-positive, strictly aerobic, and non-motile. TEM images revealed that DK17^T^ exhibited a rod–coccus morphology, with cell length of 1.1–1.7 μm and a diameter ranging from 0.8–0.9 μm. *R. jostii* RHA1 displayed a similar morphology, with cell lengths of 0.8–1.2 μm and diameters of 0.6–0.9 μm. The growth temperature range for DK17^T^ was 10–30°C (optimum 25–30°C). DK17^T^ grew within a pH range of 5.5–8.0 (optimum 7.0) and in the NaCl concentration of 0–8% (w/v) (optimum 0–6%) (**Table 1**). The growth temperature and NaCl tolerance of strain DK17^T^ are similar to those of *R. jostii* RHA1 and *R. opacus* KCTC 9811^T^. However, the pH tolerance range for *R. jostii* RHA1 and *R. opacus* KCTC 9811^T^ (5.0–9.0) is slightly broader than that of strain DK17^T^. The detailed physiological characteristics of strain DK17^T^ and closely related *Rhodococcus* strains are summarized in (**Table 1**).

**Table 1:** Differential physiological properties of the strain DK17^T^ and phylogenetically closely related *Rhodococcus* species. Strains: 1, DK17^T^; 2, *R. jostii* RHA1 [65]; 3, *R. opacus* KCTC 9811^T^ [91]; 4, *R. wratislaviensis* N805^T^ [5]; 5, *R. jostii* IFO 16295^T^ [92].

Physical characteristics	1	2	3	4	5
Source of isolation	Oil- contaminated soil	Lindane-contaminated soil	Soil around a gas pipe	Forest soil	Medieval grave
Range of temperature (optimal) °C	10-30 (25-30)	10-30 (25-30)	10-30 (25-30)	4-37 (28)	10-30 (15-30)
Range of pH (optimal)	5.5-8.0 (7.0)	5.0-9.0 (7.0)	5.0-9.0 (7.0)	4.0-9.0 (7.5)	5.0-9.0 (7.0)
NaCl tolerance (optimal) (% w/v)	0.0-8.0 (0-6)	0.0-8.0 (0-6)	0.0-8.0 (0-6)	1-6 (1)	0.0-8.0 (0-6)
**Biochemical tests**					
Oxidase	–	–	–	+	n.a.
Catalase	+	+	+	+	+
Casein	+	+	–	n.a.	–
Tween 20	+	+	–	+	n.a.
Tween 40	+	–	–	n.a.	n.a.
Tween 60	+	–	–	n.a.	n.a.
Tween 80	–	–	–	n.a.	–
Xanthine	–	+	n.a.	n,a.	+
Hypoxanthine	+	–	–	n.a.	+
**API ZYM**					
Acid phosphatase	+	+	+	n.a.	n.a.
Cystine arylamidase	+	+	+	n.a.	n.a.
α-chymotrypsin	w	–	w	n.a.	n.a.
α-galactosidase	–	–	+	n.a.	n.a.
β-galactosidae	–	–	+	n.a.	+
β-glucuronidase	–	–	–	n.a.	–
α-gulcosidase	+	+	+	n.a.	–
Lipase C14	+	+	+	n.a.	n.a.
Leucine arylamidase	+	+	+	n.a.	n.a.
Napthol-AS-BI phopshohydrolase	+	+	+	n.a.	n.a.
Trypsin	+	+	+	n.a.	n.a.
Valine arylamidase	+	+	+	n.a.	n.a.
**API 20 NE**					
Esculin ferric citrate hydrolysis	+	+	+	+	–
Urea hydrolysis	+	+	w	+	–

Characteristics are scored as: + , positive reaction; − , negative reaction; w, weakly positive reaction; n.a., not available. Data for DK17^T^, *R. jostii* RHA1, and *R. opacus* KCTC 9811^T^ are from the present study, while data for the other strains are from previously published sources.

Strain DK17^T^ along with *R. jostii* RHA1 and *R. opacus* KCTC 9811^T^ was catalase positive and oxidase negative. The strain DK17^T^ hydrolyzed the casein, hypoxanthine, Tween 20, 40, and 60. Interestingly, *R. jostii* RHA1 can hydrolyze casein, hypoxanthine, and Tween 20, whereas *R. opacus* KCTC 9811^T^ is not capable of hydrolyzing the macromolecules. In API ZYM test, all three *Rhodococcus* strains, DK17^T^, *R. jostii* RHA1, and *R. opacus* KCTC 9811^T^ showed nearly similar results. The strain DK17^T^ was positive for the activities of acid phosphatase, cysteine arylamidase, α-gulcosidase, lipase C14, leucine arylamidase, naphthol-AS-BI-phosphohydrolase, trypsin, and valine arylamidase while weak positive for α-chymotrypsin. In API 20 NE, the strain DK17^T^ was positive for the reduction of nitrates to nitrites, for the hydrolysis of esculin ferric citrate, and production of urease enzyme. However, the strain *R. opacus* KCTC 9811^T^ was only weakly positive for urease activity. Most of the physiological and biochemical characteristics determined in this study were similar between DK17^T^ and *R. jostii* RHA1. However, hydrolysis of Tween 40 and 60, which was positive in DK17^T^, was negative in RHA1 (**Table 1**).

### 3.4 Chemotaxonomic characterization

The fatty acid profile for strain DK17^T^, *R. jostii* RHA1, and *R. opacus* KCTC 9811^T^ were determined. The predominant fatty acids found in strain DK17^T^, *R. jostii* RHA1 and other closely related strains, *R. opacus* KCTC 9811^T^, *R. wratislaviensis* NCTC 13229^T^, and *R. jostii* IFO 16295^T^ were C_16:0_ and C_17:0_, ranging from 22.0–33.0% and 7.0–16.5% (**Table 2**). In DK17^T^ and *R. jostii* RHA1, C_17:1_ ω8c and summed features 3 were also predominant, ranging from 15.6–17.0% and 14.0–14.6%, respectively (**Table 2**). Additionally, only *R. jostii* RHA1 and *R. opacus* KCTC 9811^T^ possessed C_18:1_ ω9c at proportions of 14.2% and 11.0%, respectively. Notably, C_15:0_ was not detected in strains DK17ᵀ and *R. jostii* RHA1, which may serve as a distinguishing feature from other *Rhodococcus* species where this fatty acid is typically more abundant. Strain DK17^T^ possesses menaquinone 8 (MK-8) as its respiratory quinone, consistent with *R*. *jostii* RHA1 [65].

**Table 2:** Cellular fatty acid composition of the strain DK17^T^ and phylogenetically closely related *Rhodococcus* species. Strains: 1, DK17^T^; 2, *R. jostii* RHA1; 3, *R. opacus* KCTC 9811^T^; 4, *R. wratislaviensis* N805^T^; 5, *R. jostii* IFO 16295^T^. Data for DK17^T^, *R. jostii* RHA1, and *R. opacus* KCTC 9811^T^are from the present study, while data for the other strains are from previously published sources^4-5^.

Fatty acid (%)	1	2	3	4	5
**Saturated**					
C_14:0_	2.5	2.3	2		4.0
C_15:0_			**10**	**12.9**	8.0
**C**_**16.0**_	**33.0**	**29.1**	**22**	**22.6**	**29.0**
C_17.0_	**10.6**	8.9	7	**16.5**	9.0
C_18.0_		3.0	2		4.0
**Unsaturated:**					
C_16:1_ ω6c			5		
C_16:1_ ω7c			8		
**C**_**17:1**_ **ω8c**	**15.6**	**17.0**	**13**		
C_18:1_ ω9c		**14.2**	**11**		
anteiso-C_17:1_ ω9c					
**Branched:**					
C_16:1_ cis 9				5.1	
C_16:1_ cis 10				8.5	
C_17:1_ cis 9				12.6	
C_18:1_ cis 9				10.4	
C_17:0_ 10-methyl	2	2.9	6	6.3	
C_18:0_ 10-methyl		3.5	8	6.1	4.0
Summed features:*					
**3**	**14.6**	**14.0**			
6		1.0			

*Summed features represent fatty acids that could not be separated by GLC with the midi system; summed feature 3 comprises C_16:1_ ω7c and/or C_16:1_ ω6c and summed feature 6 comprises C_19:1_ ω7c and/or C_19:1_ ω11c. Fatty acids accounting for up to 10% or more are presented in bold.

### 3.5 Comparative analysis of genome functions

#### 3.5.1 Aromatic compound metabolism.

The RAST analysis showed that strain DK17^T^ carried 9,548 coding sequences (CDSs) and 61 RNAs and *R. jostii* RHA1 exhibited 9,680 CDSs and 66 RNAs. In all six genomes used for comparison, the largest number of annotated genes were assigned to amino acids and derivatives (631–778), carbohydrate metabolism (527–778), and fatty acids, lipids, and isoprenoid synthesis (300–503) (Table S3 in S2 File). Notably, all the six *Rhodococcus* strains exhibited a high number of genes related to the metabolism of aromatic compounds, with a total range of 138–177 genes identified. These genes are involved in the metabolism of various aromatic compounds such as biphenyl, benzoate, quinic acid, salicylate, gentisate, catechol, protocatechuate, and β-ketoadipate.

Strain DK17ᵀ and *R. jostii* RHA1 harbor a comparable repertoire of genes involved in the degradation of aromatic compounds, although DK17ᵀ had slightly fewer CDSs than *R. jostii* RHA1. Both strains contain genes for aromatic monooxygenases, dioxygenases, and the β-ketoadipate pathway, which are consistent with their experimentally observed ability to degrade a broad range of aromatic compounds. Among these compounds, benzoate, a simple aromatic carboxylate that is both a widespread environmental pollutant and utilizable growth substrate is metabolized by diverse microorganisms. For example, *Pseudomonas putida* KT2440 degrades benzoate through the catechol intermediates [66], while *Acinetobacter baylyi* and *Aspergillus nidulans* utilize quinate via the quinate/shikimate pathway [67,68]. In *Rhodococcus* species, benzoate is commonly degraded under aerobic conditions via benzoate 1,2-dioxygenase or benzoate monooxygenase. The benzoate-degrading ability of strain DK17ᵀ was also demonstrated in pure culture experiments [69]. These degradation processes play important roles in soil bioremediation by contributing to pollutant detoxification in terrestrial and aquatic environments [70,71]. Furthermore, the capacity to metabolize benzoate is often associated with microbial strains that can degrade structurally complex molecules, including steroids. For instance, cholesterol degradation in *Rhodococcus* begins with oxidation to cholestenone via extracellular cholesterol oxidase [72] and efficient conversion has been demonstrated in *Rhodococcus erythropolis* using alkylated cyclodextrin [73]. A cholesterol oxidase gene (*choG*) was identified in *Rhodococcus* sp. strain CECT3014 [74]. However, this gene was not detected from strain DK17^T^ and *R. jostii* RHA1, despite the confirmation of initial ring oxidation in *R. jostii* RHA1. These observations highlight the metabolic flexibility of *Rhodococcus* and their potential in bioremediation and steroid bioconversion.

Beyond biodegradation, recent studies have highlighted the ecological roles of *Rhodococcus* species in interactions with plants [75,76]. While pathogenic species such as *Rhodococcus equi* and *Rhodococcus fascians* cause foal pneumonia and leafy gall disease in plants [77,78], other strains exhibit traits associated with nitrogen metabolism and plant growth promotion. For example, *R. jostii* RHA1 encodes nitrogen lipid regulator (NlpR), suggesting regulatory capacity for nitrogen metabolism [79]. In addition, *Rhodococcus erythropolis* KB1 has been shown to synergize with *Medicago sativa* to enhance petroleum hydrocarbon degradation (up to 95%) while simultaneously promoting plant growth and nutrient enrichment [80], and *Rhodococcus qingshengii* RL1, a plant-associated strain isolated from *Eruca sativa*, carries genes enabling nitrogen fixation, phytohormone and siderophore production, and root colonization, highlighting its role in stress tolerance and plant–microbe interactions [81]. Although DK17ᵀ has not been directly evaluated for plant growth-promoting effects, its genome harbors genes encoding inorganic phosphatase (*ppx* and *ppa*) and alkaline phosphatase (*pho*D), which may support phosphate solubilization. These features, together with predicted capabilities for secondary metabolite production, provide a foundation for future studies exploring the potential of DK17ᵀ in plant–microbe interactions. Validating these metabolic traits experimentally, including biodegradation, nutrient cycling, and symbiotic interactions, will be crucial to uncovering the putative ecological functions of this strain.

#### 3.5.2 Biosynthetic gene clusters and secondary metabolite potential.

**Fig 3:**
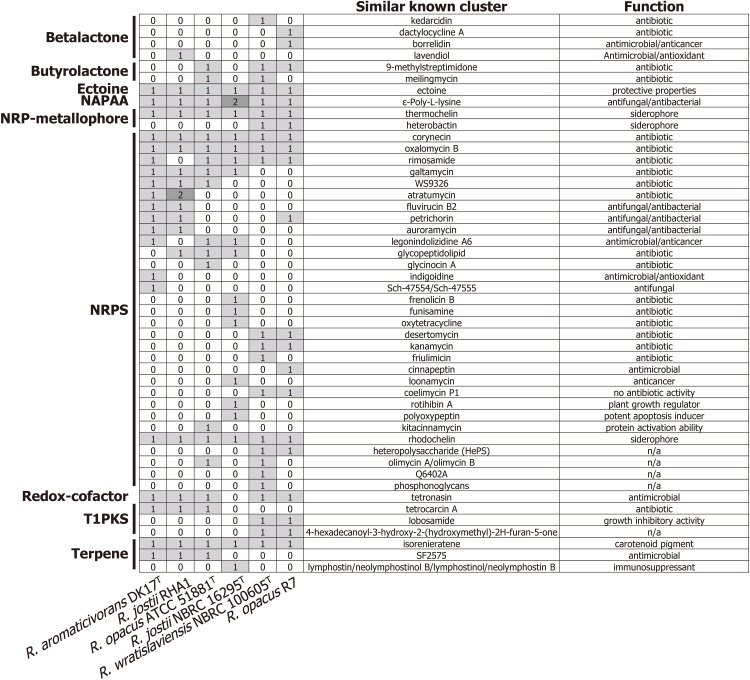
Biosynthetic gene clusters (BGCs) in *Rhodococcus* strains. NRPS, non-ribosomal peptides synthase; NAPAA, non-alpha-poly amino acid; T1PKS, type I polyketide synthase.

Analysis of biosynthetic gene clusters (BGCs) involved in the synthesis of secondary metabolites using antiSMASH revealed that the genomes of the strains used for comparison contained 18–23 BGCs (Fig 3). These BGCs belong to various groups, including non-ribosomal peptide synthases (NRPSs), polyketides, beta-lactones, and terpenes and they encode compounds that act as antibiotics, anticancer agents, antioxidants, and siderophores.

Among the BGCs, NRPSs represent biosynthetic capacity for a structurally diverse class of secondary metabolites known for their wide range of biological activities and pharmacological properties [82,83]. Strain DK17^T^ harbors NRPSs BGCs predicted to produce compounds such as atratumycin, galtamycin, indigoidine, oxalomycin B, rimosamide, and legonindolizidine A6 (Fig 3). These antibiotics represent an important group of biocontrol agents that protect plants from bacterial and fungal pathogens, thereby underscoring the significance of these strains as valuable biotechnological resources [82,84,85]. In particular, BGCs for atratumycin, fluvirucin B1, and auroramucin were found only in DK17^T^ and *R. jostii* RHA1 while other genomes did not harbor these BGCs. Interestingly, among the *Rhodococcus* strains, only DK17^T^ contains BGCs associated with fluvirucin B2, petrichorin, auroramycin, legonindolizidine A6, and indigoidine, all of which exhibit antimicrobial activity. *R. jostii* RHA1 carries a BGC predicted to encode biosynthesis of lavendiol, which functions as an antimicrobial and antioxidant compound [86]. In contrast, another group of NRPSs, including those associated with thermochelin and rhodochelin, function as siderophores that enable bacteria to sequester iron from the environment, thereby facilitating their growth under challenging conditions were found in all genomes compared [87].

The second major class of BGCs encodes the biosynthetic capacity for polyketides, which are synthesized by polyketide synthases, multi-domain enzymes responsible for producing a diverse range of secondary metabolites [88]. These BGCs encode compounds such as tetrocarcin A and lobosamide, which function as antibiotics and growth inhibitors. Furthermore, all genomes contained BGCs for isorenieratene, which are characterized by their five-carbon hydrocarbon skeleton and function as antioxidants and photoprotective pigments [89]. The results underscore the valuable contributions of DK17^T^ and related *Rhodococcus* strains to environmental and biotechnological applications through the production of significant metabolites.

### 3.6 *o*-Xylene degradation gene analysis and *in vitro* assay

**Fig 4:**
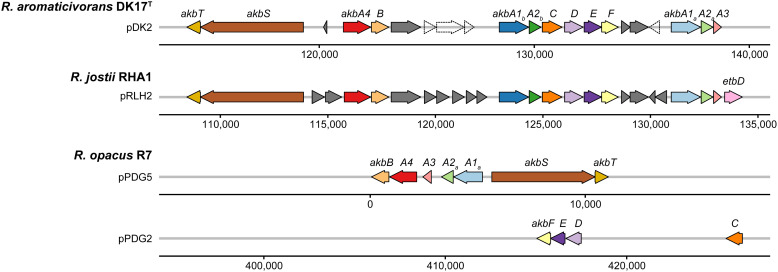
Comparison of the genetic organization of the *akb* gene cluster. The plot was visualized using the syntenyPlotteR package (v1.0.0) in R (v4.3.3). Putative genes are colored by dark gray and pseudogenes are shown in a dashed arrow shape. *akbS* and *akbT*, two-component regulatory genes; *akbA1a*-*akbA2a* and *akbA1b*-*akbA2b*, *o*-xylene dioxygenase—two sets of large and small oxygenase subunits; *akbA3*, a gene for ferredoxin component; *akbA4*, a gene for ferredoxin reductase component; *akbB*, *cis*-dihydrodiol dehydrogenase; *akbC*, meta-cleavage 2,3-dioxygenase; *akbD*, hydrolase; *akbE*, hydratase; *akbF*, aldolase.

The genus *Rhodococcus* is well-known for its ability to degrade aromatic compounds, with various strains effectively breaking down substances such as pyridine, biphenyl, and oxybenzone [14–16]. Dioxygenase plays a key role in this process by incorporating both atoms of molecular oxygen into their substrates for the degradation of aromatic compounds [90]. Strain DK17^T^ encodes a total of 76 dioxygenases or dioxygenase components, 15 of which are related to aromatic dioxygenase. Among them, four dioxygenase components (*akbA1a*, *akbA1b*, *akbA2a*, and *akbA2b* encoding aromatic ring-oxidizing dioxygenase) and one dioxygenase (*akbC* encoding aromatic ring-cleavage dioxygenase) were included in the *akb* gene cluster, indicating a role in the *o*-xylene degradation pathway. A genomic investigation of the *akb* gene cluster, an *o*-xylene degradation gene cluster, in six *Rhodococcus* species (DK17^T^, *R. jostii* RHA1, *R. jostii* NBRC 16295^T^, *R. opacus* ATCC 51881^T^, *R. opacus* R7, and *R. wratislaviensis* NBRC 100605^T^) revealed that DK17^T^, *R. jostii* RHA1, and *R. opacus* R7 harbored *akb* cluster-related genes in their genomes, while the other species did not (Fig 4). Furthermore, the *akb* cluster-related genes in pDK2 of DK17^T^ share an average amino acid sequence identity of 99.9% with those in plasmid pRLH2 of *R. jostii* RHA1. This finding suggests that the ability to degrade *o*-xylene is strain-specific within the genus *Rhodococcus.*

*In vitro* capacity for *o*-xylene degradation of strains DK17^T^, *R. jostii* RHA1, and *R. opacus* KCTC 9811^T^ was performed to confirm the involvement of *akb* cluster-related genes in the *o*-xylene degradation. The strain DK17^T^ and *R. jostii* RHA1 showed optimum growth on MSB agar plates in the presence of *o*-xylene vapor while very weak growth on MSB in the absence of *o*-xylene. The strain *R. opacus* KCTC 9811^T^ showed a very weak growth on MSB supplemented with *o*-xylene vapor. These observations are consistent with the presence of a complete *akb* gene cluster in DK17ᵀ and *R. jostii* RHA1, and its absence in *R. opacus* KCTC 9811ᵀ. Overall, our analysis highlights the important contribution of strain DK17^T^ in the degradation of various alkylbenzenes including *o*-xylene, which is crucial for bioremediation, carbon source utilization, and toxicity reduction in contaminated environments.

## 4 Conclusion

### 4.1 Description of *Rhodococcus aromaticivorans* sp. nov.

*Rhodococcus aromaticivorans* (a.ro.ma.ti.ci’vo.rans. L. masc. adj. aromaticus, aromatic, fragrant; L. pres. part. vorans, devouring; N.L. masc. part. adj. *aromaticivorans*, devouring aromatic compounds).

Cells are Gram-positive, aerobic, non-motile, catalase-positive, and exhibit a cocci-rod shape, measuring 1.1–1.7 μm in length and 0.8–0.9 μm in diameter. Colonies on R2A agar are mucoid after 48 h incubation at 30°C. The strain can grow at temperatures ranging from 10–30°C (optimum, 30°C), pH 5.5–8.0 (optimum, pH 7.0) and with 0–8% NaCl (w/v; optimum, 0–6%).

Cells are positive for the hydrolysis of casein and Tween 20 while negative for Tween 80, starch, and chitin. In the API ZYM kit, the strain was positive for the enzymatic activities of acid phosphatase, cystine arylamidase, lipase (C14), leucine arylamidase, α-glucosidase, β-glucosidase, naphthol-AS-BI-phosphohydrolase, trypsin, and valine arylamidase. In the API 20NE assay, the strain was positive for nitrate reduction, urease production, and hydrolysis of esculin ferric citrate. The predominant fatty acids found in strain DK17^T^ were C_16:0,_ C_17:1_ ω8c, and summed features 3. The respiratory quinone is MK-8.

The genomic DNA G + C content of the type strain is 67.09%. The type strain, DK17^T^ (=KCCM 90599^T^ = InaCC B1662^T^), was isolated from crude oil-contaminated site in Yeocheon, Republic of Korea. The 16S rRNA gene sequence of the strain DK17^T^ was submitted to NCBI with the GenBank accession number PQ489412. The raw sequencing reads of DK17^T^ genome have been deposited in the European Nucleotide Archive (ENA) under the accession number PRJEB81518. The genome of the strain DK17^T^ is available in the NCBI with the RefSeq assembly accession number GCF_038447505.2.

### 4.2 Reclassification of *Rhodococcus jostii* RHA1 as *Rhodococcus aromaticivorans*

Our comprehensive genome-based and physiological analyses support the reclassification of *R. jostii* RHA1, distinguishing it from the established *R. jostii*. Phylogenomic analysis revealed a strong evolutionary relationship between DK17^T^ and RHA1, with ANI (99%) and dDDH (92%) values well above species delineation thresholds, while RHA1 exhibited substantial genetic divergence from *R. jostii* DSM 44719^T^ and NBRC 16295^T^. Comparative genomic analysis further reinforced this distinction, as DK17^T^ and RHA1 shared a significantly higher proportion of genes with each other than with *R. jostii* type strains. Physiological and biochemical characteristics, including unique substrate utilization patterns and fatty acid profiles, also supported this classification. Furthermore, the *akb* cluster-related genes in plasmid pDK2 of DK17^T^ share an average amino acid sequence identity of 99.9% with those in plasmid pRLH2 of *R*. *jostii* RHA1, highlighting a close genetic relationship between these two strains in their capacity for *o*-xylene degradation. These findings provide strong evidence that RHA1 is more closely related to DK17^T^ than to *R. jostii* type strain, necessitating its taxonomic reassignment and refining the classification of these industrially significant *Rhodococcus* strains.

## Supporting information

S1 File Additional figures related to the identification of strain DK17^T^. Figure S1. Neighbor-Joining tree based on 16S rRNA gene sequences showing the phylogenetic relationship of DK17^T^ and closely related species in the genus *Rhodococcus.* Bootstrap values (>70%) in the order of NJ/ML/MP are shown at the branch points based on 1,000 replications. An asterisk (*) indicates bootstrap values below 70% in the order of NJ/ML/MP. GenBank accession numbers are shown in parentheses. Bar, 0.01 substitutions per nucleotide position. *Corynebacterium diphtheriae* NCTC 11397^T^ (X84248) was used as an outgroup. **Figure S2. Maximum likelihood phylogenomic tree inferred from concatenated alignments of 120 single-copy amino acid sequences in Genome Taxonomy Database (GTDB) (a) and Average nucleotide identity (b).** Genomic sequences were obtained from the NCBI RefSeq database under the corresponding assembly accession number. Genomes highlighted in the grey box represent non-type strains deposited under the name *Rhodococcus jostti* whereas those in the dark grey box are type strains. Bootstrap values (>70%) are indicated on the nodes. *Corynebacterium diphtheria* NCTC11397^T^ (GCF_001457455) was used as an outgroup. Scale bar, 0.05 substitutions per amino acid position.
(PPTX)

S2 File Additional tables related to DK17^T^ genome. Table S1. Summary of genome statistics. Table S3. Overview of the RAST subsystem and the numbers of genes involved in each metabolism of DK17^T^ and the closest reference strains within the genus *Rhodococcus*. Strains: 1, DK17^T^; 2, *R. jostii* RHA1; 3, *R. opacus* R7: 4, *R. opacus* ATCC 51881^T^: 5, *R. wratislaviensis* NCTC13229^T^; 6, *R jostii* NBRC 16295^T^ (DOCX)

S2 Table Additional table for DK17^T^ genome comparison. Table S2. Average nucleotide identity among *Rhodococcus* genomes. (XLSX)
