# Correction: *Rhodococcus aromaticivorans* sp. nov., an *o*-xylene degrading bacterium, and evidence supporting reclassification of *Rhodococcus jostii* RHA1

**DOI:** 10.1371/journal.pone.0342933

**Published:** 2026-02-17

**Authors:** Neak Muhammad, Jehyun Jeon, Eungbin Kim, Dockyu Kim, Yung Mi Lee

The Funding statement for this article is incorrect. The correct Funding statement is as follows: The study was supported by the Korea Polar Research Institute under Grant No. PE25140.
