# Supplementary material for: Rhodococcus aromaticivorans sp. nov., an o-xylene degrading bacterium, and evidence supporting reclassification of Rhodococcus jostii RHA1
Source: PLoS One. 2025 Dec 2;20(12):e0337194. doi: 10.1371/journal.pone.0337194 (PMC12671745; doi:10.1371/journal.pone.0337194)
Supplement: S1 File — Bootstrap values (>70%) in the order of NJ/ML/MP are shown at the branch points based on 1,000 replications. An asterisk (*) indicates bootstrap values below 70% in the order of NJ/ML/MP. GenBank accession numbers are shown in parentheses. Bar, 0.01 substitutions per nucleotide position. Corynebacterium diphtheriae NCTC 11397T (X84248) was used as an outgroup. Figure S2. Maximum likelihood phylogenomic tree inferred from concatenated alignments of 120 single-copy amino acid sequences in Genome Taxonomy Database (GTDB) (a) and Average nucleotide identity (b). Genomic sequences were obtained from the NCBI RefSeq database under the corresponding assembly accession number. Genomes highlighted in the grey box represent non-type strains deposited under the name Rhodococcus jostti whereas those in the dark grey box are type strains. Bootstrap values (>70%) are indicated on the nodes. Corynebacterium diphtheria NCTC11397T (GCF_001457455) was used as an outgroup. Scale bar, 0.05 substitutions per amino acid position. (PPTX) [file pone.0337194.s001.pptx]

## Slide 1
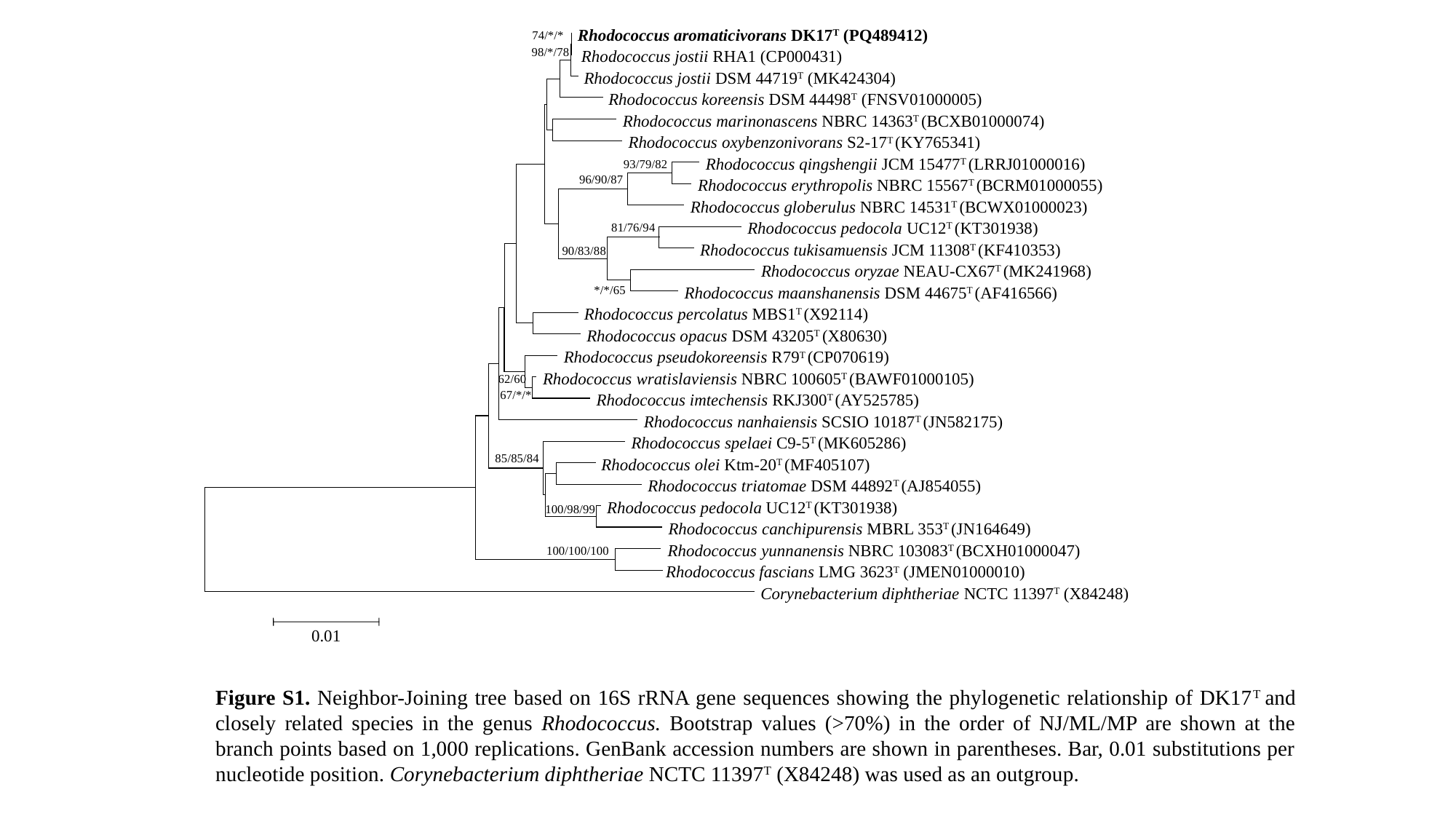

Rhodococcus aromaticivorans DK17T (PQ489412)
74/*/*
98/*/78
 Rhodococcus jostii RHA1 (CP000431)
 Rhodococcus jostii DSM 44719T (MK424304)
 Rhodococcus koreensis DSM 44498T (FNSV01000005)
 Rhodococcus marinonascens NBRC 14363T (BCXB01000074)
 Rhodococcus oxybenzonivorans S2-17T (KY765341)
 Rhodococcus qingshengii JCM 15477T (LRRJ01000016)
93/79/82
96/90/87
 Rhodococcus erythropolis NBRC 15567T (BCRM01000055)
 Rhodococcus globerulus NBRC 14531T (BCWX01000023)
 Rhodococcus pedocola UC12T (KT301938)
81/76/94
 Rhodococcus tukisamuensis JCM 11308T (KF410353)
90/83/88
 Rhodococcus oryzae NEAU-CX67T (MK241968)
 Rhodococcus maanshanensis DSM 44675T (AF416566)
*/*/65
 Rhodococcus percolatus MBS1T (X92114)
 Rhodococcus opacus DSM 43205T (X80630)
 Rhodococcus pseudokoreensis R79T (CP070619)
 Rhodococcus wratislaviensis NBRC 100605T (BAWF01000105)
62/60
67/*/*
 Rhodococcus imtechensis RKJ300T (AY525785)
 Rhodococcus nanhaiensis SCSIO 10187T (JN582175)
 Rhodococcus spelaei C9-5T (MK605286)
85/85/84
 Rhodococcus olei Ktm-20T (MF405107)
 Rhodococcus triatomae DSM 44892T (AJ854055)
 Rhodococcus pedocola UC12T (KT301938)
100/98/99
 Rhodococcus canchipurensis MBRL 353T (JN164649)
 Rhodococcus yunnanensis NBRC 103083T (BCXH01000047)
100/100/100
Rhodococcus fascians LMG 3623T (JMEN01000010)
 Corynebacterium diphtheriae NCTC 11397T (X84248)
0.01
Figure S1. Neighbor-Joining tree based on 16S rRNA gene sequences showing the phylogenetic relationship of DK17T and closely related species in the genus Rhodococcus. Bootstrap values (>70%) in the order of NJ/ML/MP are shown at the branch points based on 1,000 replications. GenBank accession numbers are shown in parentheses. Bar, 0.01 substitutions per nucleotide position. Corynebacterium diphtheriae NCTC 11397T (X84248) was used as an outgroup.

## Slide 2
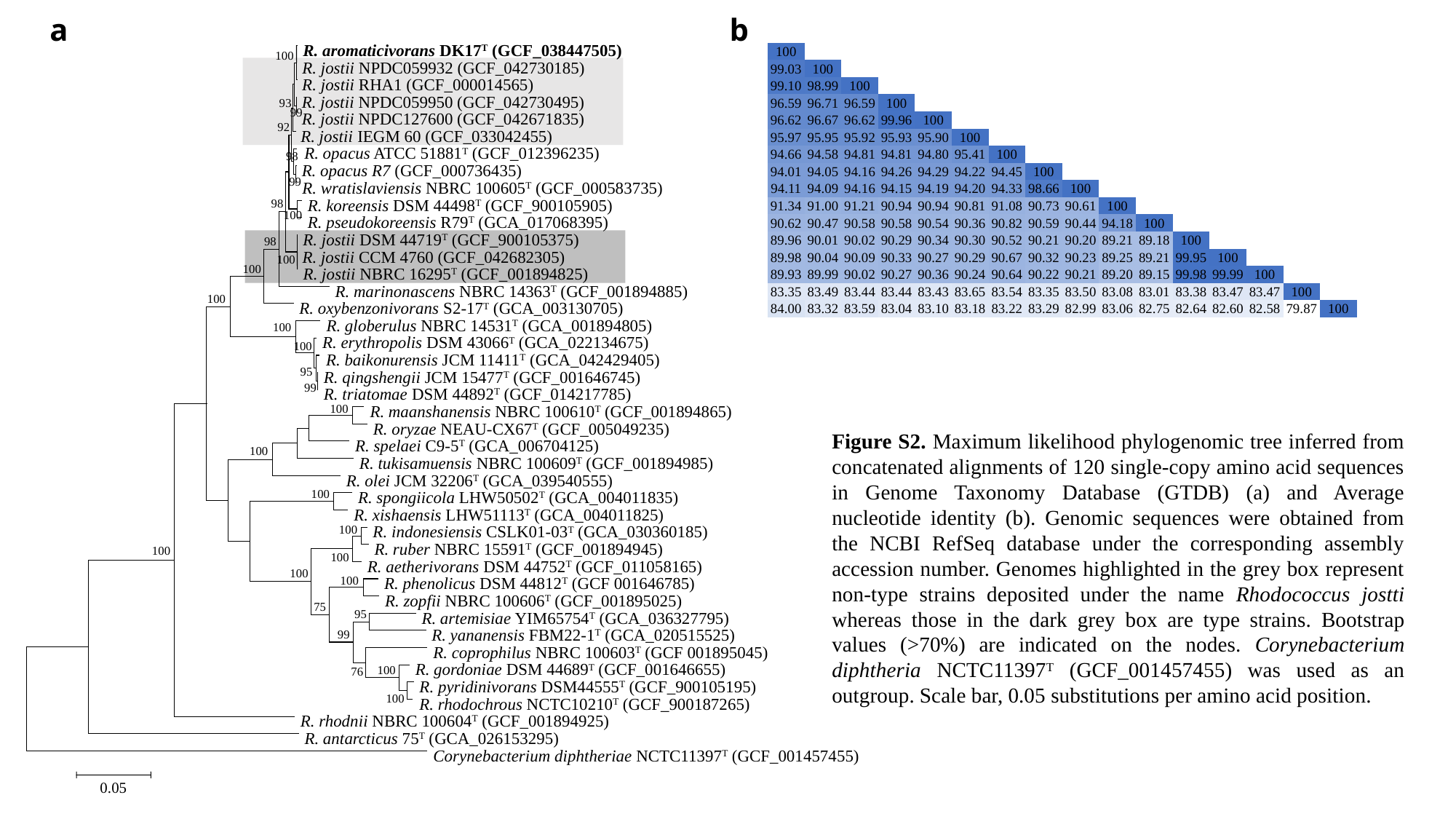

a
b
 R. aromaticivorans DK17T (GCF_038447505)
100
 R. jostii NPDC059932 (GCF_042730185)
 R. jostii RHA1 (GCF_000014565)
 R. jostii NPDC059950 (GCF_042730495)
93
99
 R. jostii NPDC127600 (GCF_042671835)
92
 R. jostii IEGM 60 (GCF_033042455)
 R. opacus ATCC 51881T (GCF_012396235)
98
 R. opacus R7 (GCF_000736435)
99
 R. wratislaviensis NBRC 100605T (GCF_000583735)
 R. koreensis DSM 44498T (GCF_900105905)
98
100
 R. pseudokoreensis R79T (GCA_017068395)
 R. jostii DSM 44719T (GCF_900105375)
98
 R. jostii CCM 4760 (GCF_042682305)
100
100
 R. jostii NBRC 16295T (GCF_001894825)
 R. marinonascens NBRC 14363T (GCF_001894885)
100
 R. oxybenzonivorans S2-17T (GCA_003130705)
 R. globerulus NBRC 14531T (GCA_001894805)
100
 R. erythropolis DSM 43066T (GCA_022134675)
100
 R. baikonurensis JCM 11411T (GCA_042429405)
95
 R. qingshengii JCM 15477T (GCF_001646745)
99
 R. triatomae DSM 44892T (GCF_014217785)
100
 R. maanshanensis NBRC 100610T (GCF_001894865)
 R. oryzae NEAU-CX67T (GCF_005049235)
 R. spelaei C9-5T (GCA_006704125)
100
 R. tukisamuensis NBRC 100609T (GCF_001894985)
 R. olei JCM 32206T (GCA_039540555)
100
 R. spongiicola LHW50502T (GCA_004011835)
 R. xishaensis LHW51113T (GCA_004011825)
 R. indonesiensis CSLK01-03T (GCA_030360185)
100
 R. ruber NBRC 15591T (GCF_001894945)
100
100
 R. aetherivorans DSM 44752T (GCF_011058165)
100
 R. phenolicus DSM 44812T (GCF 001646785)
100
 R. zopfii NBRC 100606T (GCF_001895025)
75
95
 R. artemisiae YIM65754T (GCA_036327795)
 R. yananensis FBM22-1T (GCA_020515525)
99
 R. coprophilus NBRC 100603T (GCF 001895045)
 R. gordoniae DSM 44689T (GCF_001646655)
100
76
 R. pyridinivorans DSM44555T (GCF_900105195)
100
 R. rhodochrous NCTC10210T (GCF_900187265)
 R. rhodnii NBRC 100604T (GCF_001894925)
 R. antarcticus 75T (GCA_026153295)
 Corynebacterium diphtheriae NCTC11397T (GCF_001457455)
0.05
| 100 | | | | | | | | | | | | | | | |
| --- | --- | --- | --- | --- | --- | --- | --- | --- | --- | --- | --- | --- | --- | --- | --- |
| 99.03 | 100 | | | | | | | | | | | | | | |
| 99.10 | 98.99 | 100 | | | | | | | | | | | | | |
| 96.59 | 96.71 | 96.59 | 100 | | | | | | | | | | | | |
| 96.62 | 96.67 | 96.62 | 99.96 | 100 | | | | | | | | | | | |
| 95.97 | 95.95 | 95.92 | 95.93 | 95.90 | 100 | | | | | | | | | | |
| 94.66 | 94.58 | 94.81 | 94.81 | 94.80 | 95.41 | 100 | | | | | | | | | |
| 94.01 | 94.05 | 94.16 | 94.26 | 94.29 | 94.22 | 94.45 | 100 | | | | | | | | |
| 94.11 | 94.09 | 94.16 | 94.15 | 94.19 | 94.20 | 94.33 | 98.66 | 100 | | | | | | | |
| 91.34 | 91.00 | 91.21 | 90.94 | 90.94 | 90.81 | 91.08 | 90.73 | 90.61 | 100 | | | | | | |
| 90.62 | 90.47 | 90.58 | 90.58 | 90.54 | 90.36 | 90.82 | 90.59 | 90.44 | 94.18 | 100 | | | | | |
| 89.96 | 90.01 | 90.02 | 90.29 | 90.34 | 90.30 | 90.52 | 90.21 | 90.20 | 89.21 | 89.18 | 100 | | | | |
| 89.98 | 90.04 | 90.09 | 90.33 | 90.27 | 90.29 | 90.67 | 90.32 | 90.23 | 89.25 | 89.21 | 99.95 | 100 | | | |
| 89.93 | 89.99 | 90.02 | 90.27 | 90.36 | 90.24 | 90.64 | 90.22 | 90.21 | 89.20 | 89.15 | 99.98 | 99.99 | 100 | | |
| 83.35 | 83.49 | 83.44 | 83.44 | 83.43 | 83.65 | 83.54 | 83.35 | 83.50 | 83.08 | 83.01 | 83.38 | 83.47 | 83.47 | 100 | |
| 84.00 | 83.32 | 83.59 | 83.04 | 83.10 | 83.18 | 83.22 | 83.29 | 82.99 | 83.06 | 82.75 | 82.64 | 82.60 | 82.58 | 79.87 | 100 |
Figure S2. Maximum likelihood phylogenomic tree inferred from concatenated alignments of 120 single-copy amino acid sequences in Genome Taxonomy Database (GTDB) (a) and Average nucleotide identity (b). Genomic sequences were obtained from the NCBI RefSeq database under the corresponding assembly accession number. Genomes highlighted in the grey box represent non-type strains deposited under the name Rhodococcus jostti whereas those in the dark grey box are type strains. Bootstrap values (>70%) are indicated on the nodes. Corynebacterium diphtheria NCTC11397T (GCF_001457455) was used as an outgroup. Scale bar, 0.05 substitutions per amino acid position.
