# Supplementary material for: Rhodococcus aromaticivorans sp. nov., an o-xylene degrading bacterium, and evidence supporting reclassification of Rhodococcus jostii RHA1
Source: PLoS One. 2025 Dec 2;20(12):e0337194. doi: 10.1371/journal.pone.0337194 (PMC12671745; doi:10.1371/journal.pone.0337194)
Supplement: S2 File — (DOCX) [file pone.0337194.s002.docx]

**Table S1. Summary of genome statistics**

|  | **DK17^T^** | ***R. jostii* RHA1** | ***R. opacus* R7** | ***R. opacus* ATCC 51881^T^** | ***R. wratislaviensis* NBRC 100605^T^** | ***R. jostii* NBRC 16295^T^** |
| --- | --- | --- | --- | --- | --- | --- |
| **Assembly accession number** | GCF_038447505.2 | GCF_000014565.1 | GCF_000736435.1 | GCF_012396235.1 | GCF_000583735.1 | GCF_001894825.1 |
| **Contig numbers** | 6 | 4 | 6 | 110 | 151 | 286 |
| **Contig length (bp)** | 9,527,724 | 9,702,737 | 10,118,052 | 8,111,152 | 10,403,277 | 9,727,115 |
| **Min length (bp)** | 13,770 | 332,361 | 25,175 | 1,000 | 745 | 510 |
| **Max length (bp)** | 7,909,307 | 7,804,765 | 8,466,345 | 478,617 | 484,788 | 308,680 |
| **Avg length (bp)** | 1,587,868 | 2,425,684 | 1,686,342 | 73,737 | 68,895 | 34,010 |
| **N50 (bp)** | 7,909,307 | 7,804,765 | 453,891 | 197,224 | 179,070 | 90,625 |
| **GC content (%)** | 67.09 | 66.98 | 66.71 | 67.37 | 66.78 | 66.9 |
| **Completeness (%)** | 99.59 | 99.59 | 99.59 | 99.42 | 98.92 | 98.92 |
| **Contamination (%)** | 2.01 | 0.33 | 2.7 | 1.77 | 3.03 | 1.82 |
| Each sequence length |  |  |  |  |  |  |
| Chromosome | 7,909,450 | 7,804,765 | 8,466,345 | n.d. | n.d. | n.d. |
| Linear plasmid1 | 778,981 (pDK3) | 1,123,075 (pRHL1) | 656,443 (pPDG1) | n.d. | n.d. | n.d. |
| Linear plasmid2 | 398,449 (pDK1) | 442,536 (pRHL2) | 426,388 (pPDG2) | n.d. | n.d. | n.d. |
| Linear plasmid3 | 317,199 (pDK2) | 332,361 (pRHL3) | 352,342 (pPDG3) | n.d. | n.d. | n.d. |
| Linear plasmid4 |  |  | 191,359 (pPDG4) | n.d. | n.d. | n.d. |
| Linear plasmid5 |  |  | 25,175 (pPDG5) | n.d. | n.d. | n.d. |
| Circular plasmid1 | 109,875 (pDK4) |  |  | n.d. | n.d. | n.d. |
| Circular plasmid2 | 13,770 (pDK5) |  |  | n.d. | n.d. | n.d. |

n.d.: not determined

**Table S3. Overview of the RAST subsystem and the numbers of genes involved in each metabolism of DK17^T^ and the closest reference strains within the genus *Rhodococcus*.** Strains: 1, DK17^T^; 2, *R. jostii* RHA1; 3, *R. opacus* R7: 4, *R. opacus* ATCC 51881^T^: 5, *R. wratislaviensis* NCTC13229^T^; 6, *R jostii* NBRC 16295^T^

| **Primary categories function** | **RAST subsystem category distribution** | **1** | **2** | **3** | **4** | **5** | **6** |
| --- | --- | --- | --- | --- | --- | --- | --- |
| Core Metabolic Processes | Carbohydrates | 604 | 771 | 527 | 779 | 796 | 778 |
|  | Protein metabolism | 214 | 202 | 198 | 200 | 193 | 192 |
|  | Amino acids and derivatives | 631 | 701 | 572 | 737 | 778 | 704 |
|  | Fatty acids, lipids, and isoprenoids | 341 | 420 | 300 | 441 | 503 | 424 |
|  | **Metabolism of aromatic compounds** | **169** | **165** | **138** | **177** | **177** | **163** |
|  | Respiration | 158 | 180 | 144 | 175 | 169 | 180 |
|  | Nitrogen metabolism | 52 | 40 | 31 | 36 | 39 | 38 |
|  | Phosphorus metabolism | 33 | 39 | 30 | 24 | 34 | 41 |
|  | Potassium | 13 | 10 | 9 | 8 | 8 | 10 |
|  | Sulfur metabolism | 28 | 50 | 25 | 51 | 55 | 51 |
|  | Secondary metabolism | 7 | 8 | 7 | 8 | 9 | 8 |
| Cell Structure and Genetic Function | DNA metabolism | 97 | 93 | 96 | 90 | 88 | 93 |
|  | RNA metabolism | 50 | 50 | 48 | 53 | 51 | 50 |
|  | Nucleosides and nucleotides | 127 | 141 | 136 | 143 | 154 | 137 |
|  | Membrane transport | 50 | 48 | 41 | 62 | 51 | 48 |
| Adaptation and Response | Stress response | 61 | 64 | 56 | 61 | 66 | 64 |
|  | Virulence and disease defense | 48 | 49 | 48 | 53 | 43 | 45 |
|  | Iron acquisition/metabolism | 13 | 11 | 13 | 11 | 11 | 11 |
|  | Regulation and cell signaling | 28 | 24 | 20 | 28 | 29 | 24 |
|  | Dormancy and sporulation | 1 | 1 | 1 | 1 | 1 | 1 |
